# Residential Treatment for Adolescents with Substance Use Disorders in Slovenia: A Six-Month Retrospective Preliminary Evaluation

**DOI:** 10.1192/j.eurpsy.2025.962

**Published:** 2025-08-26

**Authors:** L. Podnar Sernec, I. Kruljac

**Affiliations:** 1 Center for Treatment of Drug Addiction, University Psychiatric Clinic Ljubljana, Ljubljana, Slovenia

## Abstract

**Introduction:**

Adolescence is a critical period for the development of substance use disorders (SUD), with psychoactive substance (PAS) use posing significant mental and physical health risks. In Slovenia, the absence of specific residential treatment options for adolescents has historically limited care mostly to outpatient settings, often inadequate for severe cases. In December 2023, the University Psychiatric Clinic Ljubljana opened Slovenia’s first inpatient ward for adolescents with SUD, offering a comprehensive, structured program addressing both the psychological and physiological aspects of addiction.

**Objectives:**

The primary objectives of this study are to present the inpatient ward for adolescents with SUD program in Slovenia and to conduct an analysis of data from the first six months of the program.

**Methods:**

The program utilized a multidisciplinary approach, incorporating individual therapy, group therapy, psychoeducation, pharmacotherapy, family involvement and a range of structured therapeutic activities such as music therapy, film therapy and educational groups. A retrospective analysis of patient data from December 2023 to June 2024 was conducted. Key outcome measures included length of stay, abstinence rates, readmission rates and factors influencing premature discharge. Motivation and adherence to the therapeutic agreement were also assessed.

**Results:**

During the six-month period, 20 adolescents were admitted to the ward, resulting in a total of 32 hospitalizations, reflecting a significant rate of readmissions. The average age of the patients was 17.16 years. The length of stay varied considerably, with an average duration of 45 days. Readmissions accounted for 37.5% of total hospitalizations, often due to relapse or behavioral challenges. Motivation for treatment and adherence to the therapeutic agreement were critical factors affecting the length of hospitalization and the risk of early discharge.

**Conclusions:**

The introduction of Slovenia’s first residential treatment program for adolescents with SUD marks a significant development in the country’s healthcare system. The program offers a structured environment conducive to improving abstinence rates and addressing the complexities of adolescent substance use. However, the high rate of readmissions and the ongoing challenge of maintaining abstinence post-discharge highlight the need for refined treatment strategies, particularly regarding patient motivation and long-term relapse prevention. Future efforts should focus on optimizing the length of stay and enhancing post-discharge support to improve long-term outcomes.

**Disclosure of Interest:**

None Declared

